# Optimal exercise training for children with congenital heart disease: A systematic review

**DOI:** 10.1016/j.ahjo.2022.100119

**Published:** 2022-03-24

**Authors:** Ryo Yoshihara, Yuji Kanejima, Masahiro Kitamura, Kodai Ishihara, Kazuhiro P. Izawa

**Affiliations:** a Department of Health Science, Faculty of Medicine, Kobe University, Kobe, Japan; b Department of Public Health, Graduate School of Health Sciences, Kobe University, Kobe, Japan; c Cardiovascular stroke Renal Project (CRP), Kobe, Japan; d Department of Rehabilitation, Kobe City Medical Center General Hospital, Kobe, Japan; e Department of Physical Therapy, Fukuoka Wajiro Professional Training College, Fukuoka, Japan; f Department of Rehabilitation, Sakakibara Heart Institute of Okayama, Okayama, Japan

**Keywords:** Congenital heart disease, Exercise therapy, Health-related quality of life, Physical activity, Systematic review

## Abstract

**Background:**

Although more children with congenital heart disease (CHD) are reaching adulthood, they generally have some impairment compared to their healthy peers. Few studies have investigated the effect of exercise training on health-related quality of life (HRQOL) and/or physical activity in children with CHD.

**Purpose:**

The purpose of this study was to systematically review the effect of exercise training on HRQOL and/or physical activity and the types of training used in general.

**Methods:**

We searched relevant articles published from 2000 to 2021 in English and included intervention studies for children with CHD younger than 20 years who underwent exercise training. Afterwards, we excluded the studies not using HRQOL or physical activity as outcome measures, classified the extracted information according to outcome measures and types of interventions, and assessed the risk of bias of the included studies.

**Results:**

Finally, 10 articles were selected, and HRQOL in 3 articles and physical activity in 3 articles showed improvement after exercise training. However, 4 articles did not show improvement in these outcome measures, and 9 of the articles had a high risk of bias in blinding. Sport-based or play-based interventions were used in 5 articles, and prescribed or structured ones were used in 5 articles.

**Conclusion:**

Although exercise training for children with CHD may improve their HRQOL and/or physical activity, more studies are needed to assess the effect statistically. In children with CHD, sport-based or play-based interventions could be used as well as prescribed or structured interventions.

## Highlights

• Although more children with congenital heart disease (CHD) are reaching adulthood, they generally have some impairment.
• Although exercise training for children with CHD may improve their health-related quality of life (HRQOL) and/or physical activity.
• Sport-based or play-based interventions could be used as well as prescribed or structured interventions.

## Graphical abstract

**Figure:**
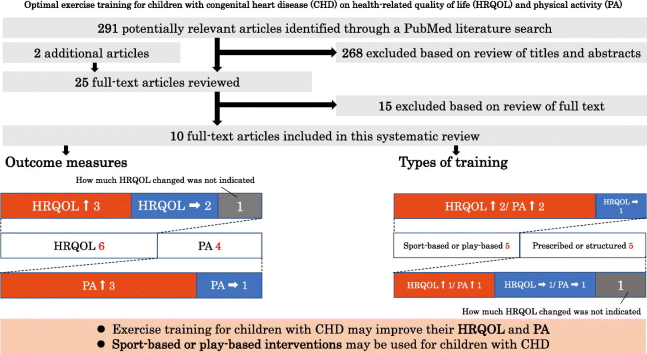

## 1 Introduction

Worldwide, data show that 13.11 in 1000 children and 6.12 in 1000 adults have congenital heart diseases (CHD) [1]. With the development of medical technology in the last few decades, >97% of children with CHD are thought to reach adulthood [2]. Despite their increased survival rates, some CHD patients have lower physical functioning, mental health, and quality of life (QOL), which are derived from the functional status related to heart defects [3]. For the purpose of improving their impairment, cardiac rehabilitation has been offered to children with CHD. However, it presents several problems such as poor adherence and difficulty in continuing regular exercise. These problems may come from the patients themselves, who are scared of exercise; their parents, who restrict their children's exercise; and a sedentary lifestyle, which leads to obesity or hypertension [4]. In general, cardiac rehabilitation consists of structured aerobic training using cycle ergometers or treadmills [5]. However, sport-based or play-based intervention is also used especially for children because playful interventions are expected to enhance their motivation [6].

A meta-analysis of CHD patients of all ages showed that exercise training has a small effect on maximal cardiorespiratory fitness, an unclear effect on health-related QOL (HRQOL), a small effect on physical activity, and a likely increase in sub-maximal cardiorespiratory fitness [7]. Moreover, a systematic review with meta-analysis suggested that exercise training should increase aerobic capacity (peak oxygen consumption) in children who have undergone surgery for CHD [8].

However, few studies have conducted a systematic review of the effect of exercise training on physical activity and HRQOL focusing especially on children with CHD (Appendix [7], [8], [9]). In addition, there is no clear guideline for exercise rehabilitation of CHD patients in Japan, and physical therapists need to carefully plan rehabilitation programs considering the patients' disease, its severity, their age, and other factors [10]. Effective exercise programs in children with CHD need to be reported so that more therapists, whether experienced or inexperienced, can offer exercise rehabilitation to these patients. Therefore, we hypothesized that exercise training interventions would more greatly improve HRQOL or physical activity in children with CHD compared to children in control groups, who live as usual or receive interventions other than exercise training. The purpose of the present study was to systematically review the effect of exercise training on physical activity and HRQOL and to investigate what types of exercise intervention were often used, focusing on children with CHD.

## 2 Methods

### 2.1 Eligibility criteria

The present systematic review was conducted based on the Preferred Reporting Items for Systematic Reviews and Meta-Analyses (PRISMA) statement [11]. We included only interventional studies in the current review. Inclusion criteria were as follows: patients younger than 20 years, intervention with exercise training, use of HRQOL or physical activity as outcome measures, published from January 2000 to September 2021, and written in English. In Japan and some other countries, the age of adulthood is 20 years. Exclusion criteria included not using either HRQOL or physical activity as outcome measures, intervention without exercise training, observational study, review, and meta-analysis.

### 2.2 Search strategy

**Fig. 1:**
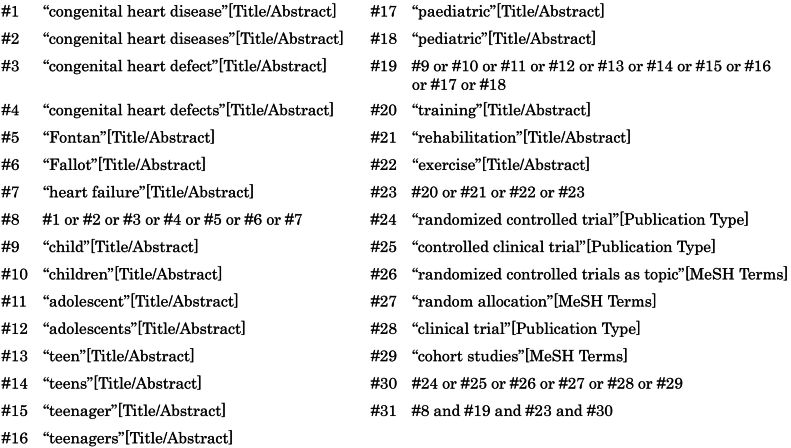
Search terms used in the current study.

Studies were searched on PubMed. The latest search was conducted on 27 October 2021. We used keywords related to “congenital heart disease”, “children”, and “clinical trials” (Fig. 1). When searching, we used filters to include only the studies published from January 2000 to September 2021. We added two articles through manual research according to the references of the article written by Williams et al. [7].

### 2.3 Selection process

**Fig. 2:**
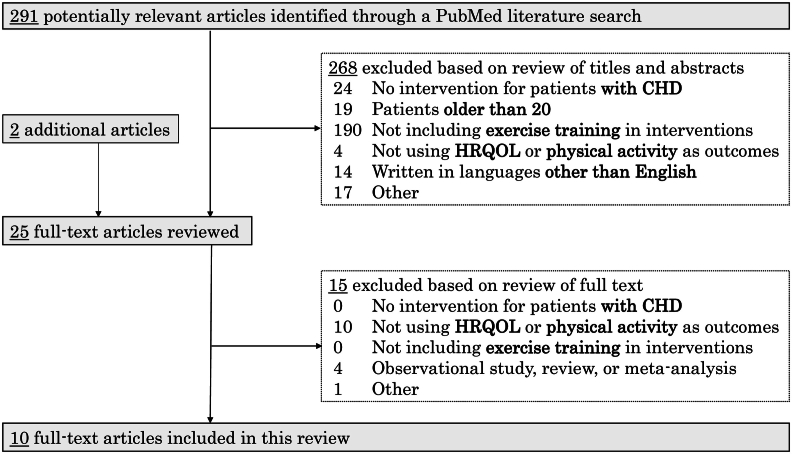
Flow diagram of this study. CHD, congenital heart disease; HRQOL, health-related quality of life.

This process consisted of title and abstract screening, full text screening, and manual research. In the title and abstract screening, the titles and the abstracts of each study were read to determine whether the study met the inclusion criteria. In the full text screening, the full text of the included manuscripts was read, and those meeting any of the exclusion criteria were excluded. When a manuscript was unavailable in our institution, we contacted the author and/or other institutions by e-mail. We sent e-mails to one author and two researchers in other institutions and obtained two articles. After the full text screening, the references of the included manuscripts were checked to search for additional potential articles. As a result, we obtained the full text of 23 articles included in the PubMed search and two articles added through manual research. This process was conducted by one researcher, and two or more collaborative researchers confirmed this method and the results. We obtained 291 relevant articles from PubMed. Following title and abstract screening, 268 articles were excluded based on the inclusion criteria. After 25 included articles were read in the full text screening, we ultimately included 10 full text articles [12], [13], [14], [15], [16], [17], [18], [19], [20], [21] in the present review (Fig. 2).

### 2.4 Data collection process

Extracted information from the included articles comprised the following: sample size, participants' age and sex, outcome measures, interventions (frequency, intensity, time, duration, and type of exercise), and results. We classified the extracted information according to outcome measures (HRQOL and physical activity) and type of interventions (sport-based or play-based intervention, and prescribed or structured intervention).

### 2.5 Risk of bias assessment

Two researchers (Y.K. and R.Y.) independently assessed the risk of bias of the included articles according to the Cochrane Collaboration Risk of Bias Tool [22], and the results from the two researchers were integrated. We evaluated each of seven domains: random sequence generation, allocation concealment, blinding (participants and personnel), blinding (outcomes assessment), incomplete outcome data, selective reporting, and other sources of bias as “low risk”, “unclear risk”, or “high risk”. Two or more collaborative researchers confirmed this method and the results.

## 3 Results

### 3.1 Study characteristics

#### 3.1.1 Overview of the included studies

We included 10 articles published in The Netherlands [13], [15], [16], [18], Germany [12], Sweden [14], the United States [17], Canada [19], Northern Ireland [20], and Norway [21]. The oldest article was published in 2000 [21]. The 10 studies consisted of eight randomized controlled trials [12], [13], [15], [16], [18], [19], [20], [21] and two non-randomized controlled trials [14], [17]. There were 766 participants in the 10 trials, and the ratio of females ranged from 24.0% to 50.0%. Most of the participants with CHD had undergone surgery, such as for the repair of Tetralogy of Fallot and single-ventricle physiology.

#### 3.1.2 HRQOL

Six of the 10 included studies used HRQOL as an outcome measure (Table 1) [12], [13], [14], [15], [16], [17]. Although two studies used the Netherlands Organization for Applied Scientific Research Academic Medical Centre (TNO/AZL) Child Quality of Life Questionnaire Child Form (TACQOL-CF) as an outcome measure [14], [15], each of the other studies used different assessment tools [12], [13], [16], [17]. Three studies reported an improvement in HRQOL [14], [16], [17], and two studies showed no improvement in HRQOL [12], [13]. One study investigated the correlation between changes in HRQOL and parental mental health or parental social support, but how much HRQOL changed was not indicated [15].

**Table 1:** Summary of articles (outcome measure: HRQOL).

Study	Sample size/ age (years)	Female ratio (%)	Outcome measures	Frequency	Intensity	Time/ Duration	Type	Results
Meyer, et al. 2021	70/ 10–18	34.2	KINDL	3/week	–	20 min/ 24 weeks	Strength and flexibility exercises	There was no improvement in total HRQOL after 24 weeks (*P* = 0.160)
van der Mheen, et al. 2019	90/ 2–8	50.0	CHQ Parent Form-50	3/week	Less than rest HR + 60–70% of HR reserve	60 min/ 3 months	Walking/jogging/running/bicycling/dynamic play	No statistically significant differences between the CHIP-Family and care-as-usual group were found in the children's outcomes
Hedlund, et al. 2018	54/ patients 14.2 ± 3.2, controls 13.6 ± 3.5	46.2	Pediatric Quality of Life Inventory Version 4.0	–	Depending on Borg scale	Depending on Borg scale/ 12 weeks	Running/jogging/skiing/cycling/riding/swimming/dancing/football	Fontan patients reported a significantly higher quality of life after training (*P* < 0.01), but the controls did not (*P* = 0.52)
Dulfer, et al. 2015	41/ 10–15	29.3	TACQOL-CF	3/week	60–70% of HR reserve	60 min/ 3 months	Aerobic dynamic cardiovascular training	Higher parental health was associated with fewer HRQOL changes in adolescents
Dulfer, et al. 2014	91/ 12.6–17.6	28.5	TACQOL-CF, TACQOL-PF	3/week	Less than rest HR + 60–70% of HR reserve	60 min/ 3 months	Walking/jogging/running/bicycling/dynamic play	Cognitive functioning improved more in the children in the exercise-group than in the control children (P < 0.05) Parents of children in the exercise-group reported improved social functioning (*P* < 0.05)
Rhodes, et al. 2006	33/ 8–17	24.3	CHQ Child Form-87	2/week	Less than HR at VAT	60 min/ 12 weeks	Stretching, aerobic, and light weight/resistance exercise	Rehabilitation patients' scores in the emotional, behavioral, and physical domains improved, whereas they declined among the control subjects

CHQ, Child Health Questionnaire; TACQOL-CF, TNO-AZL Child Quality of Life Questionnaire-Child Form; PF, Parents Form; HR, heart rate; VAT, ventilatory anaerobic threshold; HRQOL, health-related quality of life; CHIP, Child Health and Illness Profile.

#### 3.1.3 Physical activity

Four of the 10 included studies used physical activity as an outcome measure (Table 2) [18], [19], [20], [21]. All of the included studies assessed objective outcome measures with accelerometers. Although three articles measured time spent in moderate-to-vigorous physical activity, the units differed: percent [18], minutes per week [19], and minutes per day [20], respectively. Three articles reported a significant increase in physical activity [19], [20], [21].

**Table 2:** Summary of articles (outcome measure: physical activity).

Study	Sample size (age, years)	Sex (male/female)	Outcome measures	Frequency	Intensity	Time	Duration	Type	Results
Duppen et al. 2015	90 (15 ± 3)	66/24	Time spent in MVPA (%)	3/week	< (rest HR + 60–70% of HR reserve)	60 min	12 weeks	Aerobic cardiovascular training	Time spent sedentary or in moderate-to-very-vigorous activity did not change after intervention period.
Longmuir et al. 2013	61 (5.9–11.7)	36/25	MVPA (min/week)	1/week	Not indicated	Not indicated	12 months	Prescribed exercise including daily family and/or peer activity (walking, bike riding, skating)	Daily activity (MVPA) increased significantly by 6 months, decreased at 12 months, and then increased again.
Morrison et al. 2013	143 (12−20)	86/57	MVPA (min/day)	Prescribed	Prescribed	Prescribed	6 months	Prescribed	There was a significant increase in minutes of MVPA per day for the intervention group from baseline to reassessment (*P* < 0.001).
Fredriksen et al. 2000	93 (10–16)	49/44	Exercise time (*sec*), activity level (log counts)	2/week	65–80% of peak HR for at least half of physical activity time	Not indicated	2 weeks or 5 months	Swimming/football/volleyball/general activities facilitating strength, balance, coordination, flexibility, and stamina	The group undergoing training had significantly increased their level of activity (*P* = 0.028) compared to the controls (*P* = 0.053).

MVPA, moderate-to-vigorous physical activity; HR, heart rate.

#### 3.1.4 Sport-based or play-based intervention

Five of the 10 included studies used sport-based or play-based interventions [13], [14], [16], [19], [21] (Table 3). Exercise programs consisted of jogging, cycling, swimming, football, skiing, skating, and other sports. HRQOL improved in two studies [14], [16], as did physical activity in two studies [19], [21]. In one study, HRQOL did not increase [13], although the exercise program was similar to that of another study, which showed an improvement in HRQOL [16].

**Table 3:** Summary of articles (sport-based or play-based intervention).

Study	Sample size (age, years)	Sex (male/female)	Outcome measures	Frequency	Intensity	Time	Duration	Type	Results
van der Mheen et al. 2019	90 (2–8)	45/45	CHQ Parent Form-50	3/week	< (rest HR + 60–70% of HR reserve)	60 min	3 months	Walking/jogging/running/bicycling/dynamic play	No statistically significant differences between the CHIP-Family and care-as-usual group were found in the children's outcomes
Hedlund et al. 2018	54 (patients 14.2 ± 3.2, controls 13.6 ± 3.5)	29/25	Pediatric Quality of Life Inventory Version 4.0	Not indicated	Depending on Borg scale	Depending on Borg scale	12 weeks	Running/jogging/skiing/cycling/riding/swimming/dancing/football	Fontan patients reported a significantly higher quality of life after training (P < 0.01), but the controls did not (P = 0.52)
Dulfer et al. 2014	91 (12.6–17.6)	65/26	TACQOL-CF, TACQOL-PF	3/week	< (rest HR + 60–70% of HR reserve)	60 min	3 months	Walking/jogging/running/bicycling/dynamic play	Cognitive functioning improved more in children in the exercise-group than in the control children (P < 0.05) Parents of children in the exercise-group reported improved social functioning (*P* < 0.05)
Longmuir et al. 2013	61 (5.9–11.7)	36/25	MVPA (min/week)	1/week	Not indicated	Not indicated	12 months	Prescribed exercise including daily family and/or peer activity (walking, bike riding, skating)	Daily activity (MVPA) increased significantly by 6 months, decreased at 12 months, and then increased again
Fredriksen et al. 2000	93 (10–16)	49/44	Exercise time (sec), activity level (log counts)	2/week	65–80% of peak HR for at least half of physical activity time	Not indicated	2 weeks or 5 months	Swimming/football/volleyball/general activities facilitating strength, balance, coordination, flexibility, and stamina	The group undergoing training had significantly increased their level of activity (P = 0.028) compared to the controls (P = 0.053)

CHQ, Child Health Questionnaire; HR, heart rate; TACQOL-CF, TNO/AZL Child Quality of Life Questionnaire Child Form; PF, Parents Form; MVPA, moderate-to vigorous physical activity.

#### 3.1.5 Prescribed or structured intervention

Five of the 10 included studies used prescribed or structured interventions [12], [15], [17], [18], [20] (Table 4). Aerobic training was included in three studies [15], [17], [18], resistance training was included in two studies [12], [17], and flexibility exercise or stretching was included in two studies [12], [17]. Two studies showed an improvement in HRQOL or increase in physical activity [17], [20], and two studies showed no improvement [12], [18].

**Table 4:** Summary of articles (prescribed or structured intervention).

Study	Sample size (age, years)	Sex (male/female)	Outcome measures	Frequency	Intensity	Time	Duration	Type	Results
Meyer et al. 2021	70 (10–18)	46/24	KINDL	3/week	Not indicated	20 min	24 weeks	Strength and flexibility exercises	There was no improvement in total HRQOL after 24 weeks (P = 0.160)
Dulfer et al. 2015	41 (10–15)	29/12	TACQOL-CF	3/week	60–70% of HR reserve	60 min	3 months	Aerobic dynamic cardiovascular training	Higher parental health was associated with fewer HRQOL changes in adolescents
Rhodes et al. 2006	33 (8–17)	25/8	CHQ Child Form-87	2/week	< HR at VAT	60 min	12 weeks	Stretching, aerobic, and light weight/resistance exercise	Rehabilitation patients' scores in the emotional, behavioral, and physical domains improved, whereas they declined among the control subjects
Duppen et al. 2015	90 (15 ± 3)	66/24	Time spent in MVPA (%)	3/week	< (rest HR + 60–70% of HR reserve)	60 min	12 weeks	Aerobic cardiovascular training	Time spent sedentary or in MVPA did not change after the intervention period
Morrison et al. 2013	143 (12–20)	86/57	MVPA (min/day)	Prescribed	Prescribed	Prescribed	6 months	Prescribed	There was a significant increase in minutes of MVPA per day for the intervention group from baseline to reassessment (P < 0.001)

HRQOL, health-related quality of life; TACQOL-CF, TNO/AZL Child Quality of Life Questionnaire-Child Form; CHQ, Childe Health Questionnaire; HR, heart rate; VAT, ventilatory anaerobic threshold; MVPA, moderate-to-vigorous physical activity.

### 3.2 Risk of bias in the studies

**Fig. 3:**
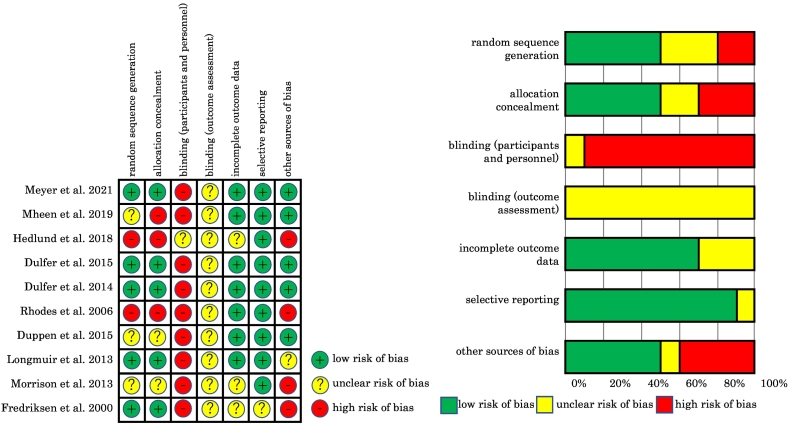
The risk of bias of each study and domain.

A summary of the risk of bias for each study and domain is shown in Fig. 3. All articles showed an unclear risk of bias in terms of blinding outcomes assessment. Most articles showed a low risk of bias in terms of incomplete outcome data and selective reporting and a high risk of bias in terms of blinding participants and personnel.

## 4 Discussion

### 4.1 Brief summary of this study

Among the included articles, six articles used HRQOL as the outcome measure, and four used physical activity. After the interventions, HRQOL improved in three articles, and physical activity improved in three articles.

HRQOL and physical activity improved after exercise training interventions in several of the studies, although most of the included studies had a high risk of bias in terms of blinding participants and personnel.

Sport-based or play-based interventions were used as often as prescribed or structured ones in the included studies.

### 4.2 Comparison with previous studies

Although Williams et al. conducted a meta-analysis of the effect of exercise on HRQOL and physical activity in CHD patients of all ages, they could find only three studies assessing HRQOL and four studies assessing physical activity [7]. In the present review, we also could not conduct a meta-analysis because we could not find enough appropriate articles; we limited the participants' age to 20 years, and each of the included studies used different outcome measures.

A previous narrative review [23] showed that most exercise training programs for pediatric CHD patients were held three times per week for 12 weeks. In the present review, the frequency and duration of training showed a little wider range than that in the previous study. This previous review [23] recommended that the intensity of training should be based on maximum heart rate. In the present review, the studies using 65–80% of peak heart rate, 60–70% of heart rate reserve, heart rate at ventilatory anaerobic threshold, or the Borg scale resulted in an improvement in HRQOL or physical activity.

In regard to training time, progressive 60-minute sessions were recommended [23]. Two studies in our review using a 60-minute exercise training program reported improved HRQOL. In addition, one study that used the Borg scale to decide training time also showed an improvement in HRQOL. Recommended programs in the previous review included aerobic activities such as running, cycling, and dance [23]. Four studies in our review used sport-based or play-based interventions such as running, skiing, and swimming and reported improved HRQOL or physical activity.

### 4.3 Possible explanations and implications

In children with CHD, sport-based or play-based interventions could be practical in some cases: e.g., children who are too small to use ergometers or treadmills, children whose adherence to exercise training is low, and children whose subjective QOL is low. As shown in the previous study [23], 60-min exercise training programs held three times per week for 12 weeks might be effective in improving HRQOL or physical activity. When deciding on the intensity of training, it might be effective to use not only maximum heart rate but any one of peak heart rate, heart rate reserve, heart rate at ventilatory anaerobic threshold, or the Borg scale.

### 4.4 Strength of the present study

To the best of our knowledge, this is the first systematic review of the effect of exercise training on the physical activity of children with CHD. We classified the articles based on the contents of the interventions (sport-based or play-based and prescribed or structured) as well as outcome measures (HRQOL and physical activity). Moreover, we explored the optimal exercise training program based on frequency, intensity, time, type, and duration of training.

### 4.5 Limitations

The present review has several limitations. First, we could not conduct a meta-analysis of both HRQOL and physical activity because we could not obtain enough articles to perform a meaningful statistical analysis. However, much data is available on exercise capacity and QOL in children and adolescents because they often participate in numerous physical activities both in and outside of school. Physical activity in everyday life may contribute to exercise capacity and QOL even if formal exercise training is difficult for children and adolescents to do. In addition, although the MOS 36-Item Short-Form Health Survey is often used to assess quality of life in adult patients [24], different outcome measures and/or units were used in the included studies of children with CHD.

Second, most of the included articles had an unclear to high risk of performance bias. This may have occurred because it was difficult to conceal from the participants the intervention groups or controls to which they were assigned. The present review contains two non-randomized controlled trials [14], [17]; however, reviewing these two trials is meaningful in that we can gain further information focused on children and adolescents.

Third, the process of screening was conducted by only one researcher, although the method and results were confirmed by two or more collaborative researchers. Therefore, incorrect exclusion of some studies might have occurred.

## 5 Conclusion

Exercise training rehabilitation for children with CHD may improve their HRQOL and physical activity. However, more studies are needed to assess the effect statistically, and future studies will need to be randomized to reduce the risk of bias in interventional studies. In addition to prescribed or structured interventions, sport-based or play-based interventions may also be used as exercise training programs for children with CHD.

## Funding

This work was not supported by any sources of funding.

## CRediT authorship contribution statement

**Ryo Yoshihara**: Conceptualization, Methodology, Investigation, Writing- Original draft, Visualization **Yuji Kanejima**: Conceptualization, Methodology, Investigation, Writing- Reviewing and Editing **Masahiro Kitamura**: Conceptualization, Methodology, Writing- Reviewing and Editing **Kodai Ishihara**: Conceptualization, Methodology **Kazuhiro P**. **Izawa**: Conceptualization, Methodology, Writing- Reviewing and Editing, Supervision, Project administration.

## Declaration of competing interest

The authors declare that they have no known competing financial interests or personal relationships that could have appeared to influence the work reported in this paper.

## Appendix A Supplementary data

Appendix Studies of effect of rehabilitation with exercise training on exercise capacity, HRQOL, and physical activity. Appendix
