# Supplementary material for: Optimal exercise training for children with congenital heart disease: A systematic review
Source: Am Heart J Plus. 2022 Mar 24;13:100119. doi: 10.1016/j.ahjo.2022.100119 (PMC10978195; doi:10.1016/j.ahjo.2022.100119)
Supplement: Appendix — Studies of effect of rehabilitation with exercise training on exercise capacity, HRQOL, and physical activity. [file mmc1.docx]

| **Appendix**  Studies of effect of rehabilitation with exercise training on exercise capacity, HRQOL, and physical activity. | | | |
| --- | --- | --- | --- |
|  | Exercise capacity | HRQOL | Physical activity |
| Children with CHD | Statistically significant increase in peak VO_2_ (Gomes-Neto et al. 2016) ^8)^ | Meta-analysis cannot be conducted due to lack of data  (Gomes-Neto et al. 2016)^8)^ | Unclear |
| Adults with CHD | Unclear | Unclear | Statistically significant increase in steps/day  (Dibben et al. 2018)^9)^ |
| All ages with CHD | Cardiopulmonary fitness may increase slightly  (Williams et al. 2020) ^7)^ | The evidence is very uncertain  (Williams CA et al. 2020)^7)^ | Physical activity may increase slightly |
| HRQOL, health-related quality of life; CHD, congenital heart disease. | | | |
